# Exposure to household air pollution and childhood multimorbidity risk in Jimma, Ethiopia

**DOI:** 10.3389/fpubh.2024.1473320

**Published:** 2024-11-01

**Authors:** Elias Mulat, Dessalegn Tamiru, Kalkidan Hassen Abate

**Affiliations:** ^1^Department of Biomedical Sciences, Institute of Health, Jimma University, Jimma, Ethiopia; ^2^Department of Nutrition and Dietetics, Food and Nutrition Research Institute, Jimma University, Jimma, Ethiopia

**Keywords:** multimorbidity, household air pollution, particulate matter, solid fuels, morbidity, children, Ethiopia

## Abstract

**Background:**

Childhood multimorbidity, characterized by the simultaneous occurrence of multiple medical conditions in children, is a global concern. Notably, exposure to household air pollution has been linked to various health issues, particularly affecting vulnerable segments of the population residing in poorly ventilated homes. However, evidence regarding the impact of household air pollution on the risk of multimorbidity in low-income settings remains scarce. Therefore, this study aims to investigate the association between household air pollution and childhood multimorbidity in Jimma, Ethiopia.

**Methods:**

A comparative cross-sectional study was conducted to collect data from 280 children under the age of five who lived in households using solid fuel (*n* = 140) and clean fuel (*n* = 140). The Demographic Health Survey morbidity questionnaire was used to collect information from mothers about common childhood illnesses. Multiple logistic regression analysis was employed to explore the relationship between the use of solid fuel for cooking in households and the likelihood of childhood multimorbidity. In addition, Poisson regression estimation was used to determine if exposure to solid fuel could increase the number of morbidities.

**Results:**

The overall prevalence of childhood multimorbidity was 34.3% [95% CI: 0.29–0.40]. Among these cases, 23.9% were among children from solid fuel user households, whereas about 10.4% were from clean fuel user households. Adjusted for all possible socioeconomic, demographic, water, sanitation, hygiene, and health care covariates, children living in solid fuel user households had more than three times the odds of childhood multimorbidity compared to children living in clean fuel user households (AOR = 3.14, 95% CI [1.42–6.95], *p* < 0.001). Moreover, household air pollution from solid fuel use was positively associated with an increased number of individual morbidity conditions, with an adjusted *β* coefficient of 0.46 (IRR = 1.58, 95% CI [1.17–2.13], *p* = 0.003).

**Conclusion:**

Solid fuel use was an independent predictor of childhood morbidity risk. Efficient policies and strategies, such as the integration of environmental regulation policies into the healthcare system aimed at the reduction of harmful air pollutants and their adverse health effects on children, need to be implemented.

## Introduction

The use of polluting biomass fuels in the kitchen is the main cause of household air pollution, which has emerged as a serious global health problem (1). The World Health Organization (WHO) estimates that over 3 billion people depend on these contaminating energy sources, and a staggering 95% of these individuals live in low- and middle-income countries (2). In this region, including Ethiopia, apart from the widespread use of biomass fuels, several factors such as poverty, substandard housing conditions, overcrowding, poor kitchen ventilation, the prevalent use of unimproved traditional cooking stoves, and cohabitation with pets and livestock in the main living room all amplify the exposure of households to air pollution (3, 4).

The incomplete combustion of biomass fuels like wood, crop residues, charcoal, and animal dung releases several harmful substances detrimental to human health (5, 6). These include carbon monoxide (CO), carbon dioxide (CO2), and fine particulate matter (PM2.5, PM10). Due to their small size, these particles have the ability to infiltrate the lungs, enter the circulatory system and reach major organs potentially resulting in acute and chronic illnesses (7, 8). The WHO attributes 6.67 million global deaths to household air pollution (HAP) related to cooking (9). This figure represents 6.7% of global mortality, surpassing the combined death tolls from malaria, tuberculosis (TB), and HIV/AIDS (10, 11). Most of this mortality is associated with respiratory infections, cardiovascular disease, low birth weight, and infant mortality (12).

Individuals of all ages are at a significant risk of severe health effects due to exposure to household air pollutants (13). In particular, children are exceptionally vulnerable to the adverse health impacts of these pollutants due to various behavioral and biological factors. When using polluting fuels for cooking, children often spend a substantial amount of time indoors, in close proximity to their mothers (14, 15). Their organs, being immature and less developed, along with their tendency to breathe, absorb, and retain more toxic substances from the air than adults, make them more susceptible to the impact of household air pollution (HAP) (16).

A recent report by the WHO states that 93% of all children, which includes 630 million under-five children, live in polluted environments around the world and are consequently exposed to unsafe levels of air pollution that exceed WHO air quality standards (13, 17). Because of the widespread use of biomass fuels for cooking, heating, and lighting in Sub-Saharan Africa, 98% of children are disproportionately affected by air pollution, putting them at higher risk for morbidity and death (18). As per a WHO report, HAP from the use of solid fuel resulted in an estimated 3.8 million premature deaths in 2016, with 543,000 of these deaths occurring among children under the age of five (3).

Air pollution is harmful at any exposure level, as low levels of pollution can hinder children’s development, increase the risk of illness, and inflict long-term damage to their immune systems, brain, lungs, and reproductive organs (19, 20). The effect of household air pollution begins early in fetal life. Exposure to household air pollution during pregnancy is linked to a 51% increased risk of stillbirths and a 38% increased risk of low birth weight (21, 22). In addition, household air pollutants increase the risk of respiratory conditions like pneumonia, allergic rhinitis, asthma, and recurrent chest infections (23). Acute lower respiratory infections (ALRI) are the second-leading cause of death for children under five, and almost all of these deaths happen in low and middle-income countries (LMICs). Moreover, air pollution causes more than half of all ALRI in children under five in LMICs (17, 24), with HAP resulting in approximately 40 million disability-adjusted life years (DALYs) and 441,000 deaths in 2016 (25).

Ethiopia bears a significant burden of illness and death among children under the age of five (13). The Ethiopian Demographic Health Survey (EDHS) revealed a 7% prevalence of acute respiratory infections (ARI) among children under five, with fever at 14% and diarrhea at 12% (26, 27). ARI, specifically pneumonia, is one of the primary causes of illness and death, accounting for 18% of all deaths (28). In Ethiopia, household air pollution (HAP) results in 50,320 deaths annually, contributing to nearly 5% of the national disease burden (26). Moreover, an Ethiopian Ministry of Health report suggests that 5% of acute upper respiratory infections and 5% of pneumonia cases, which account for 7% of hospital admissions, are believed to be linked to household air pollution (29).

Multimorbidity, the presence of more than one medical condition in a single individual, along with increased exposure to household air pollution, has become a significant public health threat in low-income countries (30, 31). In recent years, in particular, multimorbidity in children under the age of five has become an emerging public health threat in LMICs (32–36). It imposes a significant impact, including societal and economic burdens, mortality, and morbidity, and can endanger the future survival and wellbeing of children (37, 38).

Studies have shown that several individual and household-level factors, such as low socioeconomic status (39), poor water, sanitation, and hygiene conditions, and a lack of access to quality healthcare services, were consistently reported as major contributors to the high prevalence of childhood multimorbidity (35, 36, 40). However, there is sparse evidence for the potential role of household air pollution in the development of childhood multimorbidity, and the few available studies primarily focus on the relationship between exposure to household air pollution and the development of different single disease conditions (14, 41, 42). Hence, this study aims to examine the association between exposure to household air pollution and the risks of childhood multimorbidity in the study settings.

## Materials and methods

### Study design and setting

**Figure 1:**
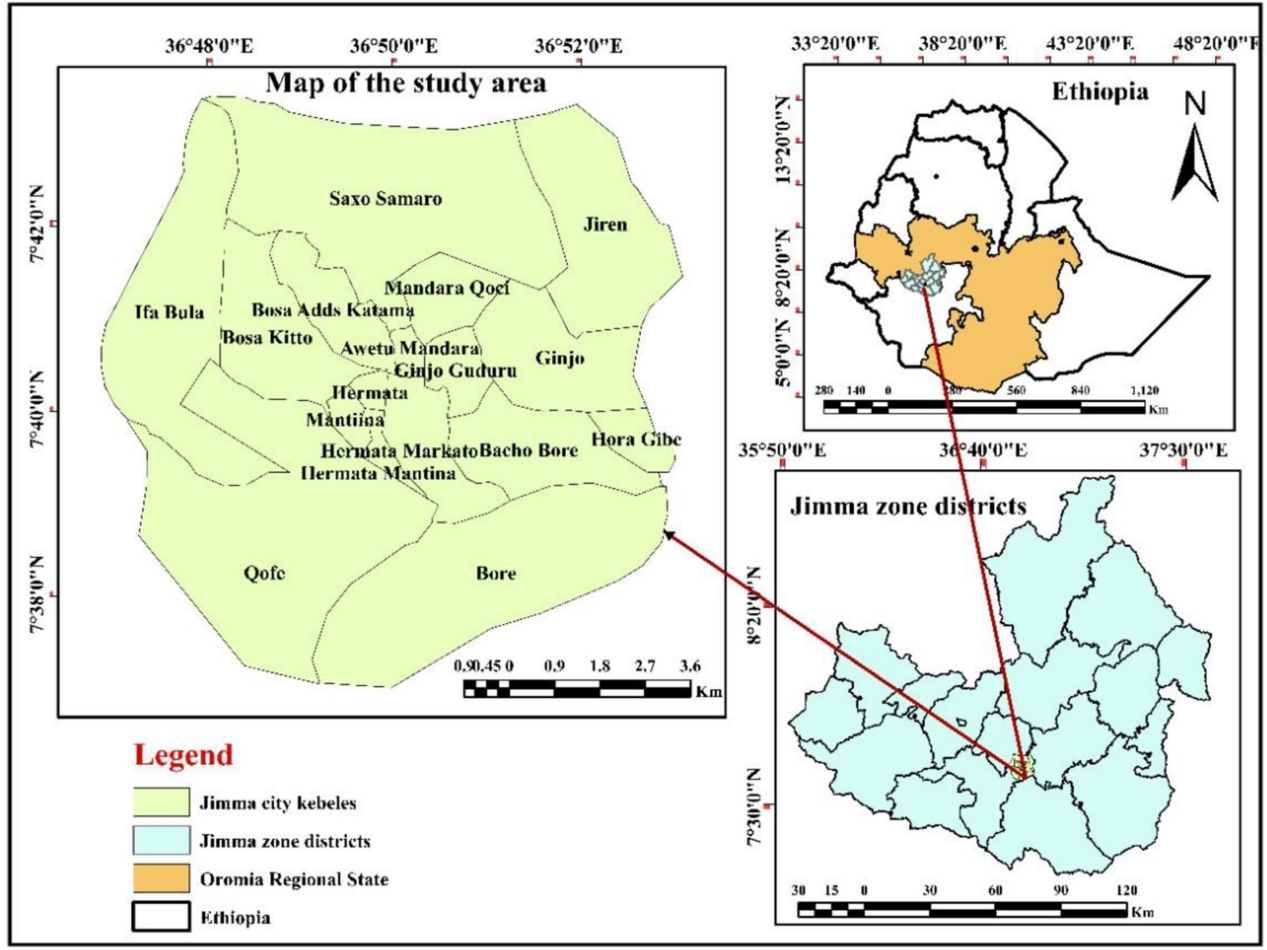
Map showing the area.

The study was conducted in Jimma town, located in the Oromia region of Ethiopia, 352 km southwest of the capital city, Addis Ababa (Figure 1). Jimma town is the capital and administrative center of the Jimma Zone. The town has an estimated population density of 239,430, divided into 12 urban and 5 semi-urban kebele (43).

### Study participants

The study included 280 under five children, 140 of them from solid fuel (wood, charcoal, crop-residues and animal dung) user households and 140 from clean fuel (electricity) user households in Jimma town Ethiopia. In the study areas, the most commonly used types of fuelwood for domestic cooking energy are branches, leaves, and twigs from species like Acacia Etbaica, Podocarpus Falcatus (yellowwood, African pine tree), and Eucalyptus (gum trees, Ironbark). The households for the study were chosen from various administrative districts/Kebeles in Jimma town, specifically Kofe, Garuke, Babala, Ginjo Guduru, and Awetu Mendera. The household selection was based on basic household fuel types used for cooking, which were the main factors in exposure to household air pollution.

### Sample size and sampling techniques

The sample size was determined using G-Power V.3.1.9.7 software, considering an equal allocation for the two groups (1:1), a 95% confidence level, 90% power, a design effect of 1.5, a medium effect size of 0.5, and a 10% nonresponse rate. Accordingly, the result yielded a total sample size of 280 children (140 from solid fuel user households and 140 from clean fuel user households). The study is part a cohort study aimed at examining the effect of HAP on children linear growth and multimorbidity (44). The initial sample size calculation takes into account the need for an adequate sample size to answer the two primary end points (linear growth and multimorbidity). As a result, we calculated different sample sizes and chose the larger sample size and greater power adequate enough to answer the study questions. Hence, we used an average HAZ score of 1.5 in exposed groups (solid fuel users) and − 1.3 in non-exposed groups (clean fuel users) from a longitudinal data analysis in LMICs (45). Study participant selections involved the selection of a study Districts and Villages from the selected Districts, followed by a selection of eligible households. Household selection was done using systematic random based on primary fuel types used for cooking and the presence of under five children. The systematic random selection method was employed to ensure a fair and unbiased selection of participants for the study. In accordance with this, first our population of interest was defined, the required sample size was determined and list households with under-five children were obtained from Health Extension workers in each district, which we entered into a random number generator in Excel. The sampling interval/fraction was then calculated using the formula *K* = *N*/*n*, and the first sampling unit was chosen randomly, followed by the selection of households every Kth interval to reach the required sample size. Finally, children under the age of five were selected from each eligible household. Eligible households were identified with the help of health extension workers in the Districts.

### Data collection procedures, techniques, and tools

#### Sociodemographic and household data

Information related to study participants’ demographics (such as age, sex, and educational status) and housing characteristics was gathered using a structured questionnaire through face-to-face interviews. Data related to socio-demographics and economic status, such as, family size, wealth index, household energy source, and cooking-related activities, were also gathered.

The Laser PM2.5 Meter-5800D/5800E was used to measure PM2.5 and PM10 concentrations, with a detection range of 0–999.9 μg/m3 and a minimum particle detection diameter of 0.3 μm. The device uses an internal laser scattering measuring principle with a relative accuracy of ±20% or ± 15 μg/m3 MAX, which can be adjusted in real-time. Similarly, Aeroqual’s TM series 500 portable air quality monitors were used to measure the levels of indoor air pollutants such as carbon monoxide (CO), carbon dioxide (CO2), and volatile organic compounds (VOC). The CO2 level was measured using a non-dispersive infrared (NDIR) sensor with a detection range of 0-2000 ppm, a minimum detection limit of 10 ppm, and a resolution of 1 ppm. Similarly, CO detection ranges from 0 to 100 ppm, with a minimum detection limit of 0.2 ppm and a resolution of 0.1 ppm. The detection range for VOC is 0–2000 PPM, with a minimum detection limit of 1 PPM at 1-PPM resolution. The monitors were calibrated to a zero filter prior to and following each sampling period (46).

#### Children’s multimorbidity status

Multimorbidity was defined as the co-occurrence of two or more diseases in the same child. The presence of the most common symptoms of childhood illnesses, including episodes of diarrhea, fever, cough, shortness of breath, runny nose, wheezing, acute respiratory infections (ARI), and skin rash, were gathered using clinical signs and symptoms from mothers’ responses in a two-week recall (47). Furthermore, multimorbidity status was constructed by counting the number of individual diseases recorded for each child for a total of eight morbidity conditions and combining these eight morbidity conditions into a count variable of multimorbidity status. Accordingly, children are categorized as those who did not experience any of the eight morbidity conditions: no condition, single condition, up to a maximum of having all eight conditions, respectively. Furthermore, the individual morbidity counts were regrouped as no multimorbidity (zero or one morbidity condition) and those with multimorbidity conditions (two or more morbidity conditions).

#### Water, sanitation, and hygiene (WASH) practice

Household drinking water sources, sanitation status, and hygiene practices were assessed using a standardized questionnaire (48). Based on the EDHS, household water sources are categorized as improved sources of drinking water, including piped water, public taps, boreholes, protected dug wells, and springs. Unimproved water sources include water from a dam, pool, or stagnant water source from a river, stream, or rainwater tank, an unprotected well, and an unprotected spring (48). Poor sanitation status is a household that has no latrine or toilet facility or a bucket system; an open latrine, an outside yard or compound, a shared private facility, an outside yard/compound, or a shared public facility. Good sanitation status is household having any non-shared toilet of the following types: flush/pour flush toilets to piped sewer systems, septic tanks, and pit latrines; ventilated improved pit (VIP) latrines; pit latrines with slabs; and composting toilets. Poor hygiene practices include individuals who have no hand washing, bathing facilities, or detergents in the house. Wash their hands with water but have no soap or other detergents. Good hygiene practices include individuals having hand washing and bathing facilities with the availability of soap and other detergents in the house (49).

#### Dietary assessment

A previously validated Food Frequency Questionnaire (FFQ) containing 28 food items most commonly consumed in the community was used to assess the minimum dietary diversity (MDD) of the children (50). The 28 food items in the food frequency questionnaire were grouped into nine food groups. A Dietary Diversity Score (DDS) was constructed by counting the intake of the food groups over 1 week and it is defined as the sum of food groups consumed over the reference period (51).

#### Data processing and analysis

Data were entered into Epi Data version 4.6 statistical software, checked for missing values and outliers, and there were no missing data. Data were exported to Statistical Package for Social Science (SPSS) version 26 for analysis. Descriptive statistics were performed to summarize the results of the outcome and independent variables using frequencies, mean, and standard deviation. Bivariate and multivariate analyses were carried out to test differences in children’s multimorbidity and households’ exposure to household air pollution. For comparison of the occurrence of multimorbidity across household fuels, different predictor variables were used: Pearson’s chi-square test for categorical variables and the Student’s t-test for continuous variables. To minimize the effect that could arise from combinations disease categories we made a rigorous data analysis first we categorized the multimorbidity as the presence of more than one disease condition and performed logistic regression and further we computed poison regression using individual disease count. Accordingly, a Poisson regression was run to predict the count of individual morbidities based on household solid fuel use. Furthermore, the individual morbidity counts were regrouped as binary outcome as no multimorbidity (zero or one morbidity condition), and those with multimorbidity conditions (two or more morbidity conditions), and multivariate logistic regression models were performed to explore the association between household fuel use and the risk of multimorbidity. Accordingly, four models were fitted to investigate the relationship: Model I was the crude model; Model II controlled for biological factors (child’s age and sex); Model III controlled for household WASH conditions based on model II; model IV further controlled for deworming, vitamin-A and iron supplementation, vaccination status, and minimum dietary diversity scores based on model III. To identify the predictors of child morbidity, a multivariable logistic regression model was fitted with child morbidity as a binary dichotomous dependent variable and other covariates. Model goodness of fit was evaluated using Hosmer and Lemeshow goodness of fit test. The results are presented as odds ratios (OR) with 95% confidence intervals to show the degree of association between dependent and independent variables at *p* < 0.05.

#### Ethics approval and consent to participate

The Jimma University Institutional Review Board (IRB) approved this study. Informed consent was obtained from a parent and legal guardian for study participation. All methods were performed in accordance with the Declaration of Helsinki.

## Results

### Demographic and socioeconomic characteristics

Two hundred and eighty children under the age of five participated in the study. Of these, 140 were from solid fuel user households, and 140 were from clean fuel user households. The mean (SD) age of children in the solid fuel group was 3.0 (1.3) years, and that of clean fuel was 3.1 (1.2) years. More than half, 52.1% of the children in the solid fuel group and 44.3% in the clean fuel group were female. The mean (SD) family size of respondents in solid fuel and clean fuel user households was 5.2 (1.8) and 4.3 (1.8), respectively. Similarly, about 37.1% of participants in solid fuel households and 28.6% in clean fuel households had a low wealth tertile. The two study groups were similar in terms of these background characteristics (Table 1).

**Table 1:** Demographic and socioeconomic characteristics of study participants, Jimma, Ethiopia, 2023.

Variables	Clean fuel, *n* (%)	Solid fuel, *n* (%)	*p*-value
Age: Mean (SD)	3.1 (1.2)	3.0 (1.3)	0.23
Sex			
Male	78 (55.7)	67 (47.9)	0.23
Female	62 (44.3)	73 (52.1)	
Wealth index			
Low	40 (28.6)	52 (37.1)	0.044*
Medium	39 (27.9)	47 (33.6)	
High	61 (43.6)	41 (29.3)	
Family size: Mean (SD)	4.3 (1.8)	5.2 (1.8)	< 0.001*
Household head			
Father	116 (82.9)	122 (87.1)	0.403
Mother	24 (17.1)	18 (12.9)	
Father education			
No formal education	17 (12.1)	42 (30.0)	< 0.001*
Primary	31 (22.1)	65 (46.4)	
Secondary	92 (65.8)	13 (23.9)	
Mother education			
No formal education	37 (26.4)	100 (71.4)	< 0.001*
Primary	58 (41.4)	38 (27.1)	
Secondary/higher	45 (32.1)	2 (1.4)	
Occupation			
Unemployed	100 (71.4)	131 (93.6)	< 0.001*
Employed	40 (28.6)	9 (6.4)	

*p* values denote *p* < 0.05* (*χ*2 test).

### Household fuel sources and pollutant concentrations

In the study, 36.4 and 13.6% of the households used wood and crop residues as the main sources of energy for cooking, respectively, while the remaining 50% used electricity as the main source of cooking energy. Additionally, about 80.7% of solid fuel user households primarily relied on traditional three stone stove while the remaining 19.3% used improved cooking stove. A statistically significant difference was observed in the concentration of indoor air pollutants between solid and clean fuel user households (*p* < 0.001) (Table 2).

**Table 2:** Household fuel sources and pollutant concentrations, Jimma, Ethiopia, 2023.

Variables		Clean fuel	Solid fuel	*p* value
Indoor air pollutants				
PM2.5 μg/m^3^	Median (IQR)	99.00 (75.80)	905.10 (336.50)	< 0.001*
	Mean rank	70.88	210.12	
PM10 μg/m^3^	Median (IQR)	119.70 (73.10)	1999 (1827.30)	< 0.001*
	Mean rank	70.95	210.50	
CO2 mg/m^3^	Median (IQR)	507.00 (123)	893.00 (1186)	< 0.001*
	Mean rank	95.64	185.36	
CO mg/m^3^	Median (IQR)	7.00 (4.60)	11.25 (20.75)	< 0.001*
	Mean rank	81.67	118.52	
VOC mg/m^3^	Median (IQR)	817 (347)	1550.50 (583)	<0.001*
	Mean rank	85.33	195.67	
T0^c^	Median (IQR)	30.0 (3.0)	29.14 (4.08)	0.029*
	Mean rank	151.01	129.99	
RH	Median (IQR)	0.43 (0.07)	0.44 (0.09)	0.495
	Mean rank	137.20	143.80	

PM2.5, Particulate matter < 2.5 μm in diameter; PM10, Particulate matter < 10 μm in diameter; CO2, carbon dioxide CO, carbon monoxide; VOC, Volatile Organic Compound; T^0c^, Temperature in degree centigrade; RH, Relative Humidity; IQR, Interquartile Range.

*p values refer to the difference between the two fuel type compared, tested with the Mann–Whitney *U* test for medians.

### Household drinking water sources, sanitation, and hygiene practices

A statistically significant difference was observed on drinking water sources, sanitation status and hygiene Practice between both group (*p* < 0.001). The majority of solid fuel user households had unimproved drinking water sources (53.6%), unimproved sanitation facilities (62.9%), and poor hygiene practices (77.9%) as compared to clean fuel user households, where about 40% of households had unimproved sanitation facilities, and 55.7 percent had poor hygiene practices.

Nearly all households (99.3%) in clean fuel households had access to improved drinking water supply from tap water piped into a dwelling, in contrast to solid fuel households, where more than half (56.3%) had no access to improved drinking water sources and depended on water supply either from an unprotected dug well, spring, or borehole. The large majority (53.6%) of solid fuel households used pit latrines, and about 22.9% of them practiced open defecation compared to their counterparts, where about 66.3% of them used pit latrines, (9.3%) composting toilets, and (7.9%) flush/pour latrines. Similarly, most of the children (60.7%) in the solid fuel group and (94.9%) in the clean fuel group commonly defecated in chamber pots, with about 39.3% of children in solid fuel households defecating in the compound or surrounding bush. Furthermore, about 49.3% of mothers from the solid fuel group and 74.3% from the clean fuel group reported washing their hands after the toilet only sometimes, and only 22.1 percent of mothers in the solid fuel group and 44.3% of the clean fuel group used soap for hand washing. Additionally, only a few (10%) mothers in the solid fuel group and (7.3%) in the clean fuel group reported washing their hands before feeding their children and preparing the children’s food. In a large proportion of the study households in both groups, utensils were washed with soap before cooking, and leftover food was commonly covered and heated before consumption. Likewise, about (20.9%) of households in the solid fuel group and 39.1% in the clean fuel group reported washing fruit and vegetables with water and salt.

### Minimum dietary diversity score, immunization status and supplementation for children

Children from solid fuel user households had a significantly low mean dietary diversity score (3.10 ± 1.28) compared to children from clean fuel user households (4.56 ± 1.30), *p* < 0.001. Furthermore, the study findings also indicated that the majority of the children from solid fuel user households had a significantly low dietary diversity score, representing 63.6% of the study respondents, compared to 19.3% of children from clean fuel user households, *p* < 0.001. Similarly, a large proportion of children in both groups had consumed more cereals, vegetables, eggs, starchy foods, and staple foods at least once over 1 week. There was a significant difference in the intake of dairy (*p* < 0.001), flesh foods (*p* < 0.001), eggs (*p* < 0.001), and fruit (*p* < 0.001) among children from clean fuel user households compared to their counterparts (Table 3).

**Table 3:** Minimum dietary diversity score and immunization status of children in Jimma, Ethiopia, 2023.

	Solid Fuel (*n* = 140)	clean fuel (*n* = 140)	
	*n* (%)	*n* (%)	*p*
Food group consumed			
Starchy and staple foods	78 (55.71)	58 (41.42)	0.023*
legumes, nuts, and seeds	127 (90.71)	66 (47.14)	< 0.001*
Dairy	43 (30.71)	103 (73.57)	< 0.001*
Flesh foods	45 (32.14)	133 (95)	< 0.001*
Eggs	125 (89.28)	140 (100)	< 0.001*
Vitamin A-rich fruit vegetables	133 (95)	138 (98.57)	0.173
Other fruits and vegetables	61 (45.57)	69 (49.28)	< 0.001*
MDD status			
Good	51 (36.4)	113 (80.7)	< 0.001*
Poor	89 (63.6)	27 (19.3)	
MDDS			
Mean (SD)	3.10 ± 1.28	4.56 ± 1.30	< 0.001**
Vitamin A supplementation			
Yes	93 (66.4)	68 (48.6)	0.004*
No	47 (33.6)	72 (51.4)	
Iron supplementation (%)			
Yes	22 (15.7)	19 (13.6)	0.735
No	118 (84.3)	121 (86.4)	
Deworming (%)			
Yes	42 (30.0)	30 (21.4)	0.132
No	98 (70.0)	110 (78.6)	
Immunization status (%)			
Fully immunized	101 (72.1)	85 (60.7)	0.101
Partially immunized	34 (24.3)	45 (32.1)	
Not immunized	5 (3.6)	10 (7.1)	

*p values denotes as *p* < 0.05* (*χ*2 test), ** *t*-test. MDDS, minimum dietary diversity score.

**Figure 2:**
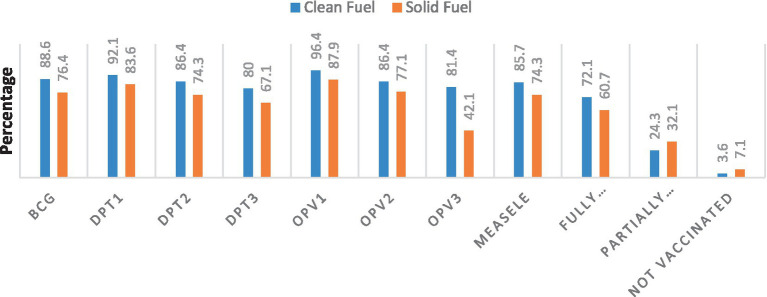
Immunization status of study participants in Jimma town, Ethiopia, 2023.

In the study, the majority (60.7%) of children in solid fuel user households and (72.1%) in clean fuel user households, were fully immunized (Figure 2).

Regarding supplementation, 48.6% of children in solid fuel and 66.4% in clean fuel user households received vitamin A supplementation; only 13.6 and 15.7% of children in solid and clean fuel user households received iron supplementation, respectively (Table 3).

### Association of household air pollution with childhood multimorbidity

The overall prevalence of childhood multimorbidity was 34.3% [95% CI: 0.29–0.40]. Of them, 23.9% were among children from solid fuel user households, whereas about 10.4% were from clean fuel user households, and the difference was statistically significant (*p* < 0.001). The most frequent causes of childhood morbidities in both groups were fever (59.3%), cough (57.1%), and diarrhea (34.3%). The proportion of acute respiratory infections (ARI) was higher among children from solid fuel user households (40%) compared with those from clean fuel user households (17.1%), and the difference was statistically significant (*p* < 0.001) (Table 4).

**Table 4:** Frequency of childhood morbidities across household fuel use, Jimma, Ethiopia, 2023.

Variables	Clean fuel	Solid fuel	*p*-value
Diarrhea (%)			
No	122 (87.1)	110 (78.6)	0.081
Yes	18 (12.9)	30 (21.4)	
Cough (%)			
No	116 (82.9)	84 (60.0)	< 0.001*
Yes	24 (17.1)	56 (40.0)	
Wheezing (%)			
No	130 (92.9)	120 (85.7)	0.082
Yes	10 (7.1)	20 (14.3)	
Shortness of breath (%)			
No	124 (88.6)	105 (75.0)	0.003*
Yes	16 (11.4)	35 (25.0)	
Runny nose (%)			
No	122 (87.1)	114 (81.4)	0.250
Yes	18 (12.9)	26 (18.6)	
Fever (%)			
No	114 (81.4)	83 (59.3)	< 0.001*
Yes	26 (18.6)	57 (40.7)	
Itchy skin rash (%)			
No	134 (95.7)	123 (87.9)	0.029*
Yes	6 (4.3)	17 (12.1)	
ARI (%)			
No	116 (82.9)	84 (60.0)	< 0.001*
Yes	24 (17.1)	56 (40.0)	
Multimorbidity status (%)			
No condition	78 (58.6)	55 (41.4)	0.001*
Single condition	18 (35.3)	33 (64.7)	
Two conditions	5 (35.7)	9 (64.3)	
Three conditions	9 (37.5)	15 (62.5)	
Four conditions	7 (31.7)	15 (68.2)	
Five and more conditions	8 (22.2)	28 (77.8)	

Values are *n* (%) unless otherwise specified; *p* values denote *p* < 0.05* (*χ*2 test).

In bivariate analysis, the use of solid fuel in households (*χ*2 = 23.43; *p* < 0.001), low education (*χ*2 = 28.32; *p* < 0.001), unemployment of mothers (*χ*2 = 8.53; *p* = 0.014), lack of vaccination (*χ*2 = 26.82; *p* = 0.001), iron (*χ*2 = 6.39; *p* = 0.041), and deworming (*χ*2 = 13.62; *p* = 0.00) for children were factors related to multimorbidity in children (Table 5).

**Table 5:** Multivariate analysis of the association between household air pollution and childhood multimorbidity risks in Jimma, Ethiopia, 2023.

Characteristics	Multimorbidity status			*p*-value
	No conditions	Single condition	Multiple conditions	
Age in years	3.2 ± 1.20	2.8 ± 1.10	2.96 ± 1.25	0.165
Sex				
Male	66 (49.6)	32 (62.7)	47 (48.9)	*χ*2 = 3.O1; *p* = 0.983
Female	67 (50.4)	19 (37.3)	49 (51.1)	
Wealth index				
Low	42 (31.6)	18 (35.3)	32 (33.3)	*χ*2 = 12.35; *p* = 0.979
Medium	39 (29.3)	24 (47.1)	23 (24.0)	
High	52 (39.1)	9 (17.6)	41 (42.7)	
Family size				
<= Five	105 (78.9)	29 (56.9)	60 (62.5)	0.095
> Five	39 (21.1)	11 (43.1)	36 (37.5)	
Father education				
No formal education	25 (18.8)	8 (15.7)	25 (26.0)	*χ*2 = 3.38; *p* = 0.092
Primary	43 (32.3)	19 (37.2)	32 (33.3)	
Secondary	65 (48.9)	23 (45.1)	67 (69.7)	
Mother education				
No formal education	44 (33.1)	28 (54.9)	65 (67.7)	*χ*2 = 28.32; *p* < 0.001*
Primary	58 (43.6)	15 (29.4)	23 (24.0)	
Secondary/higher	31 (23.3)	8 (15.7)	8 (8.3)	
Occupation				
Unemployed	103 (77.4)	40 (78.4)	88 (91.7)	*χ*2 = 8.53; *p* = 0.014*
Employed	30 (22.6)	11 (21.9)	8 (8.3)	
Fuel type for cooking				
Solid fuels	55 (41.4)	18 (35.3)	62 (64.6)	*χ*2 = 23.43; *p* < 0.001*
Clean fuels	78 (58.6)	33 (64.7)	29 (35.4)	
Water sources				
Unimproved	31 (23.3)	13 (25.5)	31 (32.3)	*χ*2 = 2.35; *p* = 0.309
Improved	102 (76.7)	38 (74.5)	65 (67.7)	
Sanitation status				
Unimproved	64 (48.1)	23 (45.1)	57 (59.4)	*χ*2 = 3.83; *p* = 0.148
Improved	69 (51.9)	28 (54.9)	39 (40.6)	
Hygiene status				
Poor	53 (39.9)	16 (31.4)	24 (25.0)	*χ*2 = 5.64; *p* = 0.060
Good	80 (60.1)	35 (68.6)	72 (75.0)	
MDD score				
Poor	48 (36.1)	20 (39.2)	48 (50.0)	*χ*2 = 5.64; *p* = 0.102
Good	85 (63.9)	31 (60.8)	48 (50.0)	
Vaccination status				
Fully vaccinated	104 (78.2)	36 (70.6)	46 (47.9)	*χ*2 = 26.81; *p* < 0.001*
Partially vaccinated	26 (19.5)	14 (27.5)	39 (40.6)	
Not vaccinated	3 (2.3)	1 (1.9)	11 (11.5)	
Vitamin A supplementation				
No	48 (36.1)	21 (41.2)	50 (52.1)	*χ*2 = 5.88; *p* = 0.053
Yes	85 (63.9)	30 (58.8)	46 (47.9)	
Iron supplementation				
No	109 (81.9)	41 (80.4)	89 (92.7)	*χ*2 = 6.37; *p* = 0.041*
Yes	24 (18.1)	10 (19.6)	7 (7.3)	
Deworming				
No	91 (68.4)	33 (64.7)	84 (87.5)	*χ*2 = 13.62; *p* = 0.001*
Yes	42 (31.6)	58 (35.3)	12 (12.5)	

Values are *n* (%) unless otherwise specified; *p* values denote *p* < 0.05* (*χ*2 test).

Multivariable logistic regression analyses were carried out to analyze the independent effects of household fuel use for cooking and the different covariates on childhood multimorbidity status. It was found that holding all other predictor variables constant, solid fuel use was positively associated with having higher odds of childhood multimorbidity as compared to children living in clean fuel user households for cooking energy (AOR = 3.14, 95% CI [1.42–6.95], *p* < 0.001, Model IV) (Table 6). Furthermore, solid fuel use significantly increased the odds of children experiencing cough (AOR = 3.22, 95% CI [1.179, 5.610], *p* < 0.001), fever (AOR = 3.0, 95% CI [1.749, 5.184], *p* < 0.001), ARI (AOR = 3.22, 95% CI [1.851, 5.610], *p* < 0.001), SOB (AOR = 2.58, 95% CI [1.354, 4.928], *p* = 0.004), and skin rash (AOR = 3.10, 95% CI [1.179, 8.081], *p* = 0.022).

**Table 6:** Logistic regression analysis of the association between household air pollution and childhood multimorbidity risks in Jimma, Ethiopia, 2023.

Morbidity status	Fuel types			
		β	$SE\left(\beta \;\right)$	OR (95% CI of OR)
Model I	Clean fuel	Ref.		1
	Solid fuel	1.256	0.269	3.513 (2.075–5.947)***
Model II	Clean fuel	Ref.		1
	Solid fuel	0.882	0.328	2.415 (1.269–4.596)***
Model III	Clean fuel	Ref.		1
	Solid fuel	1.093	0.391	2.982 (1.387–6.415)***
Model IV	Clean fuel	Ref		1
	Solid fuel	1.243	0.405	3.141 (1.419–6.952)***

***Significant at *p* < 0.001, all β coefficients (95% CI) related to the solid fuel user groups = standard error. Model 1, Unadjusted; Model 2, Biological, sociodemographic factors & wealth index; Model 3, model II plus WASH conditions; Model 4, model III plus vaccination, Deworming, Vitamin-A, and Iron supplementations, MDDS (fully adjusted). Ref, Reference category.

A Poisson regression was run to predict the number of morbidities based on household fuel types used for cooking, and the results showed that household solid fuels were positively related to children’s experiences of an increased number of morbidity conditions. Accordingly, children in households with solid fuel users have a higher risk of developing multiple morbidity conditions than children in households with clean fuel users. This is indicated by the adjusted *β* coefficient of 0.46 (IRR = 1.58, 95% CI [1.174–2.134]), which is statistically significant at *p* = 0.003 (Table 7).

**Table 7:** Poisson regression analysis of the association between household air pollution and childhood multimorbidity risks in Jimma, Ethiopia, 2023.

Models	Fuel types	Poisson regression		
		*β*-coefficient	$SE\left(\beta \;\right)$	IRR (95% CI)
Model I	Clean fuel	Ref.		
	Solid fuel	0.738	0.102	2.092 (1.712–2.55)***
Model II	Clean fuel	Ref.		
	Solid fuel	0.462	0.123	1.588 (1.247–2.021)***
Model III	Clean fuel	Ref.		
	Solid fuel	0.531	0.139	1.071 (1.294–2.236)***
Model IV	Clean fuel	Ref.		
	Solid fuel	0.459	0.152	1.583 (1.174–2.134)***

***Significant at *p* < 0.001, all β coefficients, IRR (95% CI) = Incidence risk rate were from Poisson regression analysis and related to the solid fuel user groups. SE = standard error. Model 1, Unadjusted; Model 2, Biological, sociodemographic factors & wealth index; Model 3, model II plus WASH conditions; Model 4, model III plus vaccination, Deworming, Vitamin-A, and Iron supplementations, MDDS (fully adjusted). Ref, Reference category.

## Discussion

The current study examined the impact of exposure to HAP on childhood multimorbidity risk. The overall occurrence of childhood multimorbidity in the studied children was 34.3%, and solid fuel use was found to be an independent predictor of childhood multimorbidity. Holding all other predictor variables constant, children living in solid fuel user households had higher odds of childhood multimorbidity compared to children living in clean fuel user households for cooking. Furthermore, household air pollution from solid fuel use was positively associated with an increased number of morbidities. Fever, cough, diarrhea, and ARI were observed as the most frequent causes of childhood morbidities in the study participants.

Our study results are consistent with other studies in a similar context that have found an association between household air pollution exposure and higher morbidity risks in children (52–55). Epidemiological research has demonstrated a link between exposure to household air pollution and a higher incidence of upper and lower respiratory symptoms in children, including cough, rhinorrhea, nasal obstruction, dyspnea, and wheezing (56, 57). Likewise, a meta-analysis that included 24 studies revealed that children exposed to indoor biomass fuel had a higher risk of pneumonia (OR = 1.8; 95% CI: 1.5–2.1) (58), and another meta-analysis of 25 studies confirmed a strong relationship between indoor biomass burning and acute respiratory infection in children (OR = 3.5; 95% CI: 1.9–6.4) (59). Moreover, biomass fuels were considerably associated with the development of respiratory tract infection in Ethiopia (OR 2.09; 95% CI 1.03–4.22) (29), India (OR 4.73, 95% CI 1.67–13.45) (60), and Pakistan (RR 1.5, 95% CI 1.2–1.9) (61).

Air pollution affects human health through a number of biological mechanisms. The most widely accepted theory states that oxidants and pro-oxidants in environmental pollutants when inhaled through the respiratory system, form oxygen and nitrogen free radicals, which subsequently cause oxidative stress in the airways. An increase in free radicals further initiates an inflammatory response by releasing inflammatory cells and mediators such as cytokines, chemokines, and adhesion molecules into the systemic circulation, resulting in subclinical inflammation that not only harms the respiratory system but also has systemic consequences (62–64). The alveolar space, the alveolar-capillary membrane, and the small airways are all susceptible to the effects of fine particles (PM2.5 and PM10). These particles can also undergo systemic translocation to extra-pulmonary organs, where they can enter the circulatory system (65) and eventually reach every organ system, including the kidneys, lungs, heart, and brain (66). It has also been demonstrated that exposure to air pollutants alters children’s immune systems, which suggests that exposure may increase susceptibility to microbial infections (67–69). Furthermore, Fine particulate matter can have a significant impact on children’s health, causing an inflammatory response that can spread systemically, affecting multiple organs and leading to asthma, bronchitis, and COPD. Epigenetic modifications brought on by particulate matter exposure can also impact immunological response and lung development, raising the risk encountering multiple disease. In addition, particulate matter can alter immune responses, exposing children to an increased risk of infection and chronic inflammatory diseases, which leads to multiple health conditions at the same time (70–72).

Several behavioral and physiological factors exposes children uniquely to the harmful health effects of Household air pollutants. When cooking using polluting solid fuels, children frequently spend a significant amount of time indoors, close to their mothers (14, 15). Their immature and underdeveloped organs, combined with their proclivity to breathe, absorb, and retain more toxic substances from the air than adults, make them more susceptible to household air pollution (HAP) (16, 62). Furthermore, children’s immune systems are still developing, making them more susceptible to various infections and diseases (62, 64).

The foundation for health and wellbeing is laid in early childhood, and this effect lasts throughout life. Children are regarded as the population group that requires the most protection in programs aimed at reducing the negative impact of household air pollution on health. The study’s findings provide evidence that children living in households where solid fuels are used for cooking are more likely to experience childhood morbidity than their counterparts. This implies that policymakers need to consider the impact of indoor air pollution on childhood morbidity and develop effective intervention strategies to reduce exposure to health-damaging indoor air pollutants. Most importantly, it provides insight into a more comprehensive strategy that will address the root causes and viable solutions that significantly reduce the burden of childhood morbidity. This helps national efforts to meet SDG-related targets like clean air, energy, and health and wellbeing. Additionally, the study establishes the basis for future research on the relationship between solid fuel use and the risks of childhood morbidity.

The use of biomass fuels in developing countries is expected to stay stable or even rise in the near future due to a number of challenges in obtaining clean energy fuel, such as cost, accessibility, availability, supply, and demand (73). Additionally, using inefficient cooking appliances and having inadequate ventilation in the kitchen increases the likelihood of exposure to harmful air pollutants. This is especially true for young children, particularly for young children who suffer high rates of exposure when their mothers cook while caring for them on their backs, significantly increasing their risk of morbidity. Thus, it is critical to develop effective policy and intervention plans that reduce the harmful effects of HAP on human health. Different intervention approaches, including the provision of low-cost, improved cooking stoves, improving kitchen ventilation, individual behavior changes to avoid exposure through education on the negative impact of HAP, and the importance of keeping children away while cooking, can significantly reduce their exposure to health-damaging pollutants, thereby reducing childhood morbidity risk.

### Strengths and limitations of the study

The study’s strength is that it attempted to measure household air pollution quantitatively at first. It also investigated the effects of HAP on multiple disease conditions, as few studies have used DHS data to investigate the effects of HAP on single disease conditions, most notably respiratory infections. The study also included children from households that used clean fuel as a comparison group. The nature of the study design, however, limited the study because it assessed the exposure and the outcome simultaneously and was limited to one study area. Additionally, in the chi-squared test, we combined adjacent cells with zero disease counts to achieve an adequate sample size necessary for a valid analysis. This could lead to some short coming such as, obscuring specific pattern of information, less sensitive to differences between groups, and reduces the degree of freedom which can affect the tests power and interpretation of results which is reflected as a limitation of the chi-squared test is that it requires large sample size.

## Conclusion

Solid fuel use was an independent predictor of childhood morbidity risk. Children living in households that used solid fuel for cooking had a higher prevalence of childhood multimorbidity risk compared with children living in households that used clean fuel for cooking. Additionally, maternal education, vaccination status, Iron supplementations and deworming also reduce the risks of childhood multimorbidity. Hence, to mitigate the impact of HAP on children’s health, a multifaceted approach is necessary. This includes the promotion of clean cooking technologies, improved home ventilation, and the use of cleaner fuels. Public health interventions must also focus on educating communities about the risks associated with HAP and the benefits of transitioning to cleaner alternatives. Furthermore, policy initiatives aimed at reducing HAP must be integrated into broader strategies for sustainable development. Effective policies and strategies, such as integrating environmental regulation policies into the healthcare system aimed at reducing harmful air pollutants and their adverse health effects on children, need to be implemented.

## Data Availability

The original contributions presented in the study are included in the article/supplementary material, further inquiries can be directed to the corresponding author.
